# Delineating a reference interval for functional alpha-1 antitrypsin in the serum of healthy Pi*MM donors using an anti-neutrophil elastase capacity assay

**DOI:** 10.1186/s12931-026-03705-3

**Published:** 2026-07-09

**Authors:** Keith David Merdek, Dobrin Draganov, Yong Kim, Maria Laura Sargentini-Maier, Nathan Thibault, James Kalabus, Varun Ramani, Carson Veldstra, Michelle Lee

**Affiliations:** 1 Sanofi, Cambridge, MA USA; 2 Sanofi, Paris, France; 3 Sanofi, Ghent, Belgium; 4 Inhibrx Biosciences, Inc, San Diego, CA USA; 5 Formerly Inhibrx Biosciences, Inc, San Diego, CA USA; 6 Sanofi, Morristown, NJ USA

**Keywords:** Alpha-1 antitrypsin, Alpha-1 antitrypsin deficiency, Chronic obstructive pulmonary disease, Emphysema, Reference interval

## Abstract

**Background:**

Alpha-1 antitrypsin deficiency (AATD) is a genetic disease resulting in decreased circulating functional levels of alpha-1 antitrypsin (AAT), an important protease inhibitor that protects tissue, particularly the lungs, from protease-mediated degradation. Individuals with AATD-associated emphysema have historically been treated with plasma-derived AAT, requiring weekly infusions. A recombinant AAT-Fc fusion protein, efdoralprin alfa, is currently being studied in clinical trials. Here, we sought to delineate a normal range of functional AAT (fAAT) that can be used to assess how effectively therapies restore fAAT to the normal range.

**Methods:**

Self-reported healthy individuals from Massachusetts were recruited to serve as donors to delineate a reference interval (RI), with requirements for ≥ 75% of individuals to be White. The Mayo Clinic analyzed blood samples from each donor to confirm wildtype Pi*MM phenotype and assess total AAT protein levels using the A1ALC test and a Siemens nephelometry assay, respectively. Samples were assessed for fAAT at BioAgilytix using a validated anti-neutrophil elastase capacity assay with WHO International AAT standards. Verification cohorts were recruited from geographically distinct regions (Florida and California) to confirm that the Massachusetts cohort was representative of the broader population.

**Results:**

Of 237 individual donors in the RI cohort, 211 with confirmed wildtype Pi*MM phenotype were used to calculate the RI. The central 95% range (2.5th percentile to 97.5th percentile) of fAAT concentration was 23.8–42.4 µM, with 23.8 µM designated as the lower limit of normal (LLN). Although one of the Florida verification cohorts was not included because of inability to verify sample-handling procedures, the LLN determined from the RI cohort was in alignment with the other two verification cohorts. In addition, the lower bound for total AAT protein from the RI cohort was 106 mg/dL. This value was consistent with two larger published datasets, supporting the representativeness of the RI cohort.

**Conclusions:**

The fAAT RI and LLN delineated by this study can be applied directly to clinical trials to evaluate the pharmacodynamic effect of AATD therapies, and, in conjunction with clinical trial pharmacokinetic data, can be used to support dosing decisions for patients with AATD.

## Background

Alpha-1 antitrypsin (AAT) is a potent inhibitor of neutrophil elastase and other proteases [1, 2]. Alpha-1 antitrypsin deficiency (AATD) is a genetic disease resulting in insufficient levels of functional AAT (fAAT), which can lead to protease-mediated damage to the lungs, liver, skin, and blood vessels, increasing the risk of chronic obstructive pulmonary disease (COPD), emphysema, and liver disease [1]. AATD is caused by pathogenic variants in the *SERPINA1* gene and is estimated to affect 1 out of 2500 people (of European descent) in the United States, Australia, and Europe. Approximately 1 to 3% of the population with COPD are thought to have AATD [3–7].

The protease inhibitor (Pi) M allele is associated with normal AAT levels and activity; however, pathogenic variants can result in moderate to severe decreases in total AAT protein levels and/or function. Small changes to the AAT amino acid sequence resulting from genetic variants can cause defects in AAT protein folding, leading to accumulation in hepatocytes and causing chronic liver disease, and reduced affinity for neutrophil elastase, leading to unchecked protease-mediated degradation of lung tissue, causing lung disease [2, 8]. A marked reduction in circulating levels of AAT has traditionally been the hallmark of classifying the severity of AATD; however, total AAT protein levels alone may provide an incomplete description of the condition, as protein can be nonfunctional [8].

Quantitative methods such as nephelometry have been used to assess the degree of AAT insufficiency, but such measures represent total AAT levels as opposed to fAAT, which can provide more physiologically relevant information [8–11]. Using only total AAT measures to determine the risk of disease can potentially lead to inappropriate treatment decisions and increased healthcare expenditure [11]. Here, a reference interval (RI) and a lower limit of normal (LLN) for fAAT in serum of individuals with the normal Pi*MM phenotype were determined and verified. This RI is intended to delineate the normal physiological range of fAAT in healthy individuals, which can be used as a benchmark when assessing how effectively plasma-derived AAT and investigational therapies restore patient fAAT levels to within the normal range.

## Methods

### Participant population

The collection of serum from self-reported healthy donors from Massachusetts was commissioned by Inhibrx (San Diego, CA) for the purpose of delineating an RI for fAAT in normal individuals with Pi*MM phenotype. Serum collection was contracted through Precision for Medicine at their Food and Drug Administration-registered donor facilities in Norton, MA, and Mansfield, MA. Race was self-reported by donors; however, ≥ 75% of donors were required to be White based on the predominance of AATD in Northern European populations. All donors consented to an institutional review board (IRB)-approved biospecimen procurement protocol prior to donation.

To demonstrate validity and assess the lack of geographical bias of the RI delineated from this initial population, samples from three additional cohorts (FL1, FL2, CA3) representing two geographically distinct regions, Florida (FL) and California (CA), were collected, phenotyped to determine *SERPINA1* allele variants, and analyzed for fAAT concentrations. The biospecimen procurement vendor for these collections was BioIVT (West-Sussex, United Kingdom). Recruitment for FL1, FL2, and CA3 was performed in October 2023, February 2024, and February-March 2024, respectively. For these cohorts, race distribution was not controlled, and all donors consented to an IRB-approved biospecimen procurement protocol prior to donation. The FL1 dataset was ultimately rejected due to inability of the vendor to confirm proper sample handling.

### Serum collection, phenotyping, and AAT protein concentration analysis

Whole blood was collected from each donor, clotted, processed into serum, and immediately frozen to -80 °C. Serum from each individual was sent to specialized laboratories for phenotype verification, total AAT protein level determination, and fAAT measurements. Donor samples were phenotyped at the Mayo Clinic Laboratories (MCL; Rochester, MN) using the AAT proteotype S/Z, liquid chromatography-tandem mass spectrometry (LC-MS/MS) serum A1ALC test in order to identify and exclude donors with *SERPINA1* pathogenic variants from RI and LLN determinations. Only donors with the *SERPINA1* wildtype phenotype Pi*MM were used to calculate the RI and LLN. The RI was defined as the values between the 2.5th and 97.5th percentile, based on guidance from the Clinical Laboratory Standards Institute [12, 13].

Serum total AAT protein levels were measured at MCL using a Siemens nephelometry assay (Siemens, Inc., Newark, DE). This Siemens nephelometry assay was also validated at another contract research organization called QPS labs (QPS; Groningen, Netherlands), where extended sample stability assessment for AAT protein in serum was performed. All samples were measured within the stability window established during validation of this assay at QPS.

Functional AAT levels were quantified at BioAgilytix Labs (Durham, NC) using a validated anti-neutrophil elastase capacity (ANEC) assay. This method utilized the World Health Organization (WHO) AAT International Standard, sourced from the National Institute for Biological Standards and Control, to prepare the calibration standards [14]. Method validation and sample analysis were conducted using a single lot of WHO AAT reference standard. To increase the precision of the RI, each donor sample was analyzed two independent times, with coefficient of variation up to 20% between sample replicates considered acceptable. The average back-calculated concentration of the two independent results for each donor was used for calculating the RI and LLN.

### Statistical analysis

Distribution of fAAT across RI and verification groups was analyzed using an unpaired, nonparametric Mann-Whitney test. A *P*-value < 0.05 was considered statistically significant. Statistical comparisons of fAAT levels across the Pi*MM, Pi*MS, Pi*MZ, and Pi*SZ phenotypes were not formally analyzed for significance, as this was not the intention of the study. GraphPad Prism Version 9.4.0 (GraphPad Software, Boston, MA) or later was used for statistical analyses.

## Results

### Demographics

The RI cohort consisted of 237 individuals, with 80 recruited in November and December 2021, and an additional 157 recruited in March and April 2023. The verification cohorts each included 60 individuals, for a total of 417 donor samples collected across the four cohorts (RI, FL1, FL2, CA3). Of these, 374 had the Pi*MM phenotype: RI, *n* = 211; FL1, *n* = 57; FL2, *n* = 52; CA3, *n* = 54. Among Pi*MM donors, the mean age was 46.2, with 53% identifying as female and 47% as male (Table 1), and across the three cohorts in which race was recorded (RI, FL2, CA3), 63% were White, 22% were Black, 10% were Other, and 4% were Asian. In the RI cohort, 76% of Pi*MM donors were White.

**Table 1:** Demographics of Pi*MM individuals and all Pi* allele individuals across the reference interval and verification cohort participants

Available donor demographics	Pi*MM donors (*n* = 374)	All donors (*n* = 417)
Female	198 (53%)	213 (51%)
Male	176 (47%)	204 (49%)
Age, years		
Mean (SD)	46.2 (15.8)	46.2 (15.9)
Median (min, max)	47 (18, 92)	47 (18, 92)
Race^a^	(*n*=317^a^)	(*n*=357^a^)
White	200 (63%)	233 (65%)
Black	69 (22%)	73 (20%)
Other (mixed, multiracial)	30 (10%)	32 (9%)
Asian	14 (4%)	15 (4%)
Unknown/declined to answer	4 (1%)	4 (1%)

*FL* Florida, *SD *standard deviation

^a^Race was not recorded for participants in the FL1 cohort (*n* = 60); therefore, for racial demographics: Pi*MM donors, *n* = 317; all donors, *n* = 357

### Total serum AAT concentration

For the RI cohort, the median total AAT protein concentration from Pi*MM donors (*n* = 211) was 136 mg/dL with a central 95% range (2.5th to 97.5th percentile) of 106–190 mg/dL. To examine whether this cohort was representative of donors nationwide, these data were compared to data from two large, previously published AAT protein concentration datasets from Pi*MM donors (Table 2). One dataset (Donato, *N* = 17,355 Pi*MM donors), with measurements also performed by MCL using the Siemens immune nephelometric assay, reported a median total AAT protein concentration of 149 mg/dL and a central 95% range of 100–273 mg/dL [15]. Another dataset (Bornhorst, *N* = 58,087 Pi*MM donors), with measurements collected using a similar immunoturbidimetric assay from Roche, found the median total AAT protein concentration to be 147 mg/dL and the central 95% range to be 102–254 mg/dL [16]. Because the main goal of the present study was to delineate an LLN of fAAT using the RI cohort, the agreement in the lower bound (2.5th percentile) of observed total AAT protein concentrations was considered sufficient and supportive evidence that the RI cohort for this study was representative of the nationwide donors included in the two larger datasets, despite the observed difference in the upper bound.

**Table 2:** Summary of AAT protein concentrations in reference interval cohort Pi*MM individuals compared to nationwide datasets of Pi*MM individuals

					AAT Dataset Statistics	
Source	Individuals	Phenotype	*N*	Assay type	Median	95% range (2.5th -97.5th )
Bornhorst et al.	Observed - adults	Pi*MM	58,087	Immunoturbidimetric assay	147 mg/dL	102–254 mg/dL
Donato et al.	Observed - adults	Pi*MM	17,355	Immunonephelometric assay	149 mg/dL	100–273 mg/dL
Reference interval	Healthy - adults	Pi*MM	211	Immunonephelometric assay	136 mg/dL	106–190 mg/dL

*AAT *alpha-1 antitrypsin

### Functional AAT concentration

**Fig. 1:**
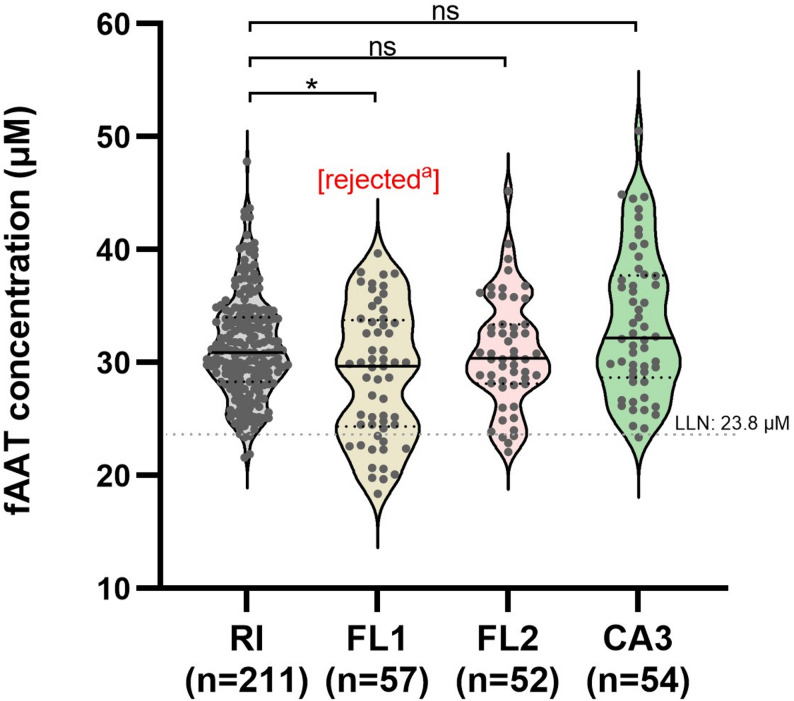
Distribution of functional AAT in Pi*MM donors by cohort. ^a^The FL1 dataset was rejected as non-validated due to inability of the vendor to confirm that supplied instructions for blood collection, processing, and handling of serum samples were followed. *P = 0.009, calculated using an unpaired, nonparametric Mann-Whitney test. ns, *P* ≥ 0.05. Dotted gray line represents the LLN from the RI cohort. Solid black lines represent median fAAT value in each sample set; dotted black lines represent Q1 and Q3. AAT, alpha-1 antitrypsin; CA, California; fAAT, functional AAT; FL, Florida; LLN, lower limit of normal; ns, not significant; Q1, quartile 1; Q3, quartile 3; RI, reference interval

The median fAAT concentration from the Pi*MM donors of the RI cohort was 30.9 µM. The central 95% range (2.5th to 97.5th percentile) was 23.8–42.4 µM, with the lower bound (2.5th percentile) of 23.8 µM designated as the LLN. Compared to the RI cohort, verification donor cohorts FL2 and CA3 independently demonstrated similar fAAT concentration distributions with no statistically significant differences in median (30.4 µM and 32.2 µM for FL2 and CA3, respectively) and central 95% range (22.4–43.7 µM and 23.7–48.4 µM, respectively) (Fig. 1; Table 3). Coefficient of variation for all duplicate fAAT sample values was ≤ 18.4%, which was within the pre-specified acceptable range.

**Table 3:** Descriptive summary of functional AAT levels across cohorts (Pi*MM individuals)

Functional AAT levels (µM)	Reference interval cohort (*n* = 211)	Verification Datasets		
		FL1 cohort (*n* = 57) [rejected^a^]	FL2 cohort (*n* = 52)	CA3 cohort (*n* = 54)
Median (min, max)	30.9 (21.6, 47.8)	29.7 (18.4, 39.7)	30.4 (22.1, 45.2)	32.2 (23.4, 50.5)
Range	26.2	21.3	23.1	27.1
2.5th percentile	23.8	19.0	22.4	23.7
97.5th percentile	42.4	38.9	43.7	48.4
Mean	31.4	29.0	30.9	33.4
SD	4.579	5.79	4.88	6.375
SEM	0.315	0.766	0.676	0.868

*AAT* alpha-1 antitrypsin, *CA* California, *FL* Florida, *SD* standard deviation, *SEM* standard error of the mean

^a^The FL1 dataset was rejected as non-validated due to inability of the vendor to confirm that supplied instructions for blood collection, processing, and handling of serum samples were followed

The vendor for the FL1 cohort could not confirm that supplied instructions for blood collection, processing, and handling of serum samples were followed; thus, the FL1 dataset was rejected as non-validated and potentially compromised in sample quality. The FL2 cohort was ordered to replace the FL1 cohort. The vendors for the FL2 and CA3 cohorts confirmed that supplied instructions for sample collection, processing, and handling were followed. This could explain the statistically significant lower distribution (central 95% range: 19.0-38.9 µM, median: 29.7 µM) observed for the FL1 cohort.

**Fig. 2:**
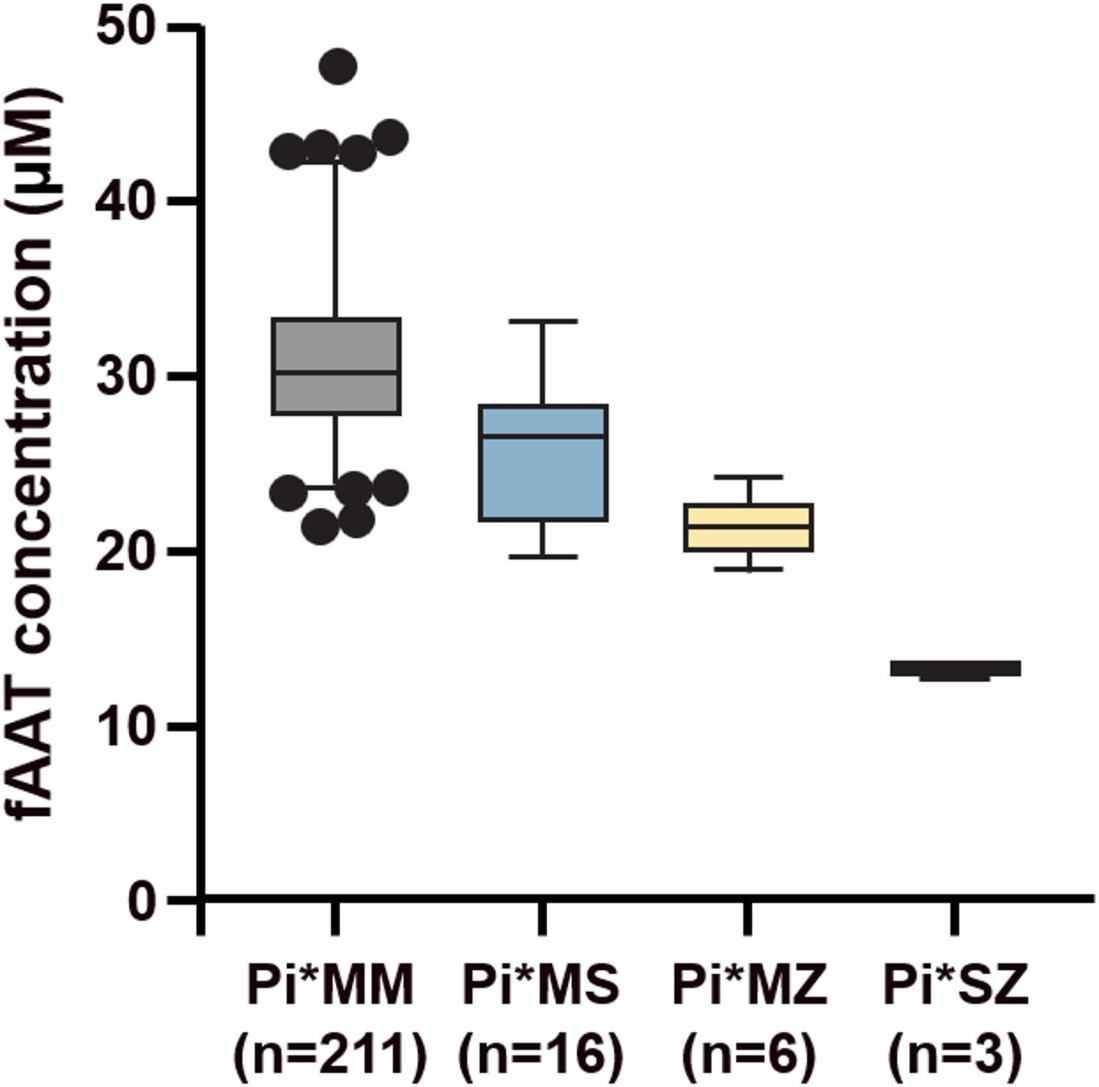
Distribution of fAAT levels by Pi* phenotype in the reference interval cohort. Box represents median with Q1, Q3; whiskers indicate 2.5th and 97.5th percentiles. fAAT, functional alpha-1 antitrypsin; Q1, quartile 1; Q3, quartile 3

Although most donors from the RI cohort exhibited the wildtype Pi*MM phenotype (*n* = 211), the remainder exhibited other phenotypes. We compared the fAAT concentrations of individuals with the Pi*MM phenotype to those exhibiting pathogenic variant phenotypes Pi*MS (*n* = 16), Pi*MZ (*n* = 6), and Pi*SZ (*n* = 3). Although the fAAT concentration for each of the non-Pi*MM phenotypes was numerically lower than that of the Pi*MM phenotype, the low sample sizes of the pathogenic phenotypes precluded any formal statistical comparison (Fig. 2). Despite the limited data available, the relationships in decreasing levels of measured fAAT in pathogenic AATD variants follow the trends of decreased total AAT previously reported with these phenotypes [15].

## Discussion

Evaluating therapeutic efficacy across clinical studies is reliant upon the bioanalytical methods used to test study samples, especially for quantitative methods where results are directly related to the specific reference standard used in the analytical method. Although measurement of total serum AAT protein is an important diagnostic tool in AATD, fAAT measurement may provide more physiologically relevant information to evaluate the effect of therapies. For example, pathogenic variants of *SERPINA1* (e.g., the F allele) demonstrate normal or near-normal levels of circulating AAT protein, but these forms of AAT exhibit deficiency in their ability to inhibit neutrophil elastase, predisposing these individuals to lung diseases [5].

In this study, a normal reference range and LLN for fAAT was delineated and verified. Based on the RI determined in individuals with confirmed wildtype Pi*MM phenotype, the central 95% of AATD-unaffected individuals have fAAT concentration levels between 23.8 and 42.4 µM, with only 2.5% having fAAT levels below 23.8 µM, designated as the LLN.

To assess whether the RI cohort from Massachusetts was representative of a broad population of donors in the United States, we compared the lower bound of total serum AAT protein from subjects with wildtype Pi*MM phenotype from the RI cohort to that of subjects with wildtype Pi*MM phenotype from two other large datasets. Because those studies had much larger numbers of participants, the closeness in lower bound for total serum AAT concentrations among the three studies supports the notion that the cohorts measured in this RI study are representative of the broader donor population from the United States. Moreover, we confirmed this observation by comparing fAAT concentrations from the RI cohort to those of individuals from geographically distinct cohorts from Florida and California. The lack of a statistically significant difference in LLN for concentrations of fAAT between the RI cohort and verification cohorts supports the validity of the LLN and its applicability as a benchmark level of fAAT in healthy, AATD-unaffected, Pi*MM individuals.

Questions may arise about the comparison between the RI delineated here for fAAT levels and established normal ranges for total AAT protein levels. Several large studies have been performed to establish normal reference ranges for AAT protein levels [15–17]. These studies used slightly differing immunoassay methods, such as immunonephelometry and immunoturbidity, and employed unique reference standards. Brantly et al. reported that 20 µM to 53 µM is the central 90% range for total AAT protein levels in Pi*MM individuals using a nephelometry-based method with a standard prepared from AAT protein purified from plasma and characterized in house [17]. While Siemens manufacturer standards were employed in the study by Donato et al., and Roche reference standards were used in the study by Bornhorst et al., neither study specifies the reference standard that these company-specific standards were calibrated against [15, 16]. It is unlikely that the aforementioned reference standards from these previous studies were the WHO International AAT reference standard, which was generated with the intent of assessing *functional* activity, as was done in this study [14]. Thus, comparisons between prior reported total AAT protein normal ranges and the fAAT RI determined here should be avoided, as discordance is to be expected between assays drawing quantitative results using different calibration or reference standards. This is further compounded by the use of completely different methodologies (functional enzymatic assay vs. immunonephelometry and immunoturbidity), which are subject to inherent assay-related specificity and sensitivity differences [9, 18].

This study was not without limitations. The sample size precluded statistical analysis of fAAT concentrations between individuals with the wildtype Pi*MM phenotype and those with AATD phenotypes Pi*MS, Pi*MZ, and Pi*SZ (Fig. 2). However, as the donor population comprised self-reported healthy individuals, the inclusion and analysis of individuals with such rare deficiencies was not a primary aim of the study. Additionally, it could also be argued that the requirement for ≥ 75% of total participants in the RI cohort to be White could bias the results. However, the robust Bornhorst study reported serum concentrations of total AAT by race, with no meaningful differences observed in the total AAT median and lower bound between White (*n* = 5,813), Black (*n* = 791), or Hispanic (*n* = 270) donors [16]. Importantly, only the RI cohort required ≥ 75% of participants to be White; there were no race requirements placed on the verification cohorts. Among the Pi*MM donors across the RI, FL2, and CA3 verification cohorts, 63% were White. Despite the lower percentage of White participants across the verification cohorts, the median and LLN of fAAT were similar between the RI and verification cohorts, therefore demonstrating the unlikeliness that controlling for race in the RI cohort affected the LLN that this study defined.

## Conclusion

The delineation of a normal range of fAAT in individuals with wildtype Pi*MM phenotype may help to evaluate the effectiveness of current and future AATD therapies in bringing fAAT into the normal physiological range. Additionally, it may provide a unique opportunity for objective direct head-to-head comparison of standard plasma-derived and various engineered and structurally differentiated recombinant protein products, and may be used to support dosing decisions in clinical studies and clinical development of potential AATD therapies. To enable such comparison to the LLN for the fAAT concentration delineated here, the same assay type (ANEC) and the same reference standard (WHO International AAT) must be used during the clinical study bioanalysis.

## Data Availability

Qualified researchers may request access to datasets used and/or analysed during the current study. Patient-level data will be anonymized, and study documents will be redacted to protect the privacy of study participants. Further details on Sanofi’s data sharing criteria, eligible studies, and process for requesting access can be found at [https:/www.vivli.org](https:/www.vivli.com) .

## Abbreviations

AAT: Alpha-1 antitrypsin
AATD: Alpha-1 antitrypsin deficiency
ANEC: Anti-neutrophil elastase capacity
fAAT: Functional alpha-1 antitrypsin
LLN: Lower limit of normal
RI: Reference interval
WHO: World Health Organization
